# Antibody–Drug Conjugate Stability Probed by Variable-Temperature Electrospray Ionization Mass Spectrometry

**DOI:** 10.1021/jasms.5c00109

**Published:** 2025-05-23

**Authors:** Jan Fiala, Dina Schuster, Albert J. R. Heck

**Affiliations:** 1 Biomolecular Mass Spectrometry & Proteomics, Bijvoet Center for Biomolecular Research & Utrecht Institute for Pharmaceutical Sciences, Utrecht University, Padualaan 8, 3584 CH Utrecht, The Netherlands; 2 Netherlands Proteomics Center, Padualaan 8, 3584 CH Utrecht, The Netherlands

## Abstract

Antibody–drug conjugates (ADCs) are effective anticancer biotherapeutics, often referred to as “magic bullets” due to their high specificity and cytotoxicity. This unique drug class consists of cytotoxic drugs coupled to monoclonal antibodies that target antigens on cancer cell surfaces. Different modes of drug conjugation are used to produce ADCs, whereby it has been shown that the employed linkage chemistries influence the drug load distribution as well as the stability of the product. While different methods to assess ADC stability are available, they mostly assess bulk properties and thus fail to assess stabilities at an individual stoichiometric drug-load level. Here, we demonstrate that variable-temperature electrospray ionization mass spectrometry can be used to study the heat stability of antibody–drug conjugates, resolving distinct stabilities for individual drug-loaded variants. As this stability is a key attribute of ADCs, we propose that variable-temperature electrospray ionization mass spectrometry may become an asset in the toolbox of analytical chemistry approaches to characterize ADCs in molecular fine detail.

## Graphical abstract

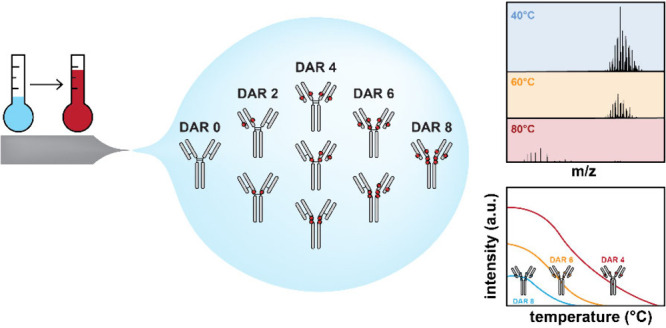

## Introduction

Antibody–drug conjugates represent a promising class of anticancer therapeutics. Their unique design combines high tumor-targeting specificity with the potency of cytotoxic antitumor compounds that are often too toxic for standalone administration. Their mechanism of action relies on the release of the cytotoxic drug after internalization into the target cancer cells.^1–3^ To date, the FDA has approved 13 different ADCs, with 6 of them targeting hematological cancers and the other 7 targeting solid tumors.^4^ A very recent approval was granted for datopotamab deruxtecan in early 2025.

Structurally, ADCs consist of three components: a monoclonal antibody, a chemical linker, and a small molecule drug as cytotoxic payload. The composition of these components varies, with differences in the antibody backbone, the employed linker chemistries, and the drug attachment chemistries. To increase the stability in systemic circulation, ensure sufficient engagement of innate immune cells, and reduce the risk of hypersensitivity against ADCs,^5^ most of the currently approved ADCs (except for 2 IgG4κ ADCs) are based on IgG1κ monoclonal antibodies.^6^ Of the currently approved ADCs, 4 are lysine-conjugated and 9 are cysteine-conjugated, with most of them possessing cleavable (rather than noncleavable) linkers. The employed conjugation strategy influences the amount of payload that can be linked to the monoclonal antibody. The typically achievable drug–antibody ratios (DARs) of lysine-conjugated ADCs are in the range of 2–5, and the DARs for cysteine-linked ADCs are between 4 and 8. This variation in DARs leads to substantial heterogeneity and makes batch-to-batch consistency in ADC production a challenge.^7,8^

Drug conjugation to an antibody can alter its physical stability and thereby its safety profile.^9,10^ While higher DARs can elicit higher antitumor efficacy, they tend to be less stable and less tolerable and their clearance rates become increased.^11–14^ For the development of cysteine-linked ADCs the antibody interchain disulfide bonds need to be (partly) reduced and modified.^15^ Beckley et al. reported already that fully loaded cysteine-conjugated ADCs (DAR 8) are less stable than the low DAR or its unconjugated counterparts. They concluded that the modification of hinge-disulfides affects the antibody’s C_H_2 domain stability and leads to increased unfolding and aggregation.^16^ Gandhi et al. showed that lysine-conjugated ADCs are more prone to aggregation induced by agitation or heat than their unconjugated precursor antibodies, potentially due to increased hydrophobic contacts and charge neutralization.^17,18^

Because of the manufacturing process, ADCs are mostly heterogeneous mixtures of antibodies with different payload distributions, varying extents of chemical modifications and variations in glycosylation.^19,20^ Due to their physical, chemical, and structural differences, the stabilities of each ADC species contained in a final ADC product may vary. Physical instability of ADCs can render them suboptimal or even unsuitable for *in vivo* applications. To adequately assess the stability of therapeutics, appropriate and reliable analytical methods are essential. Commonly employed methods to characterize the stability of ADCs include differential scanning calorimetry, size exclusion chromatography, UV–Vis spectroscopy, circular dichroism spectroscopy, dynamic light scattering, capillary isoelectric focusing, hydrophobic interaction chromatography, and surface plasmon resonance, among others.^10,21^ While these methods provide insights into diverse indicators of stability, they often analyze the ADCs as a single product, ignoring the potential unique features of individual ADC isomers harboring distinct drug-loads.

Mass spectrometry (MS)-based methods have been gaining in popularity for the characterization of biotherapeutics, due to their unique analytical sensitivity, specificity, resolving power, as well as the ability to study complex samples, and the potential to be combined with various separation and detection techniques.^19,22^ While bottom-up approaches can be used for purity assessment or site-specific conjugation stability, intact and top-down methods have shown their utility for the assessment of DAR values.^23–26^ Native mass spectrometry facilitates the study of spatial conjugation arrangement, as well as stoichiometry, gas-phase stability, structural heterogeneity, and charge distribution of ADCs.^27–31^ More recently, variable temperature electrospray ionization mass spectrometry (vT-ESI-MS) has been demonstrated and used to study structural transitions of proteins in solution,^32–37^ including the heat-stress behavior of biopharmaceutically relevant proteins.^38^ The method can be applied to nonmodified immunoglobulins and has been shown to faithfully recapitulate thermal conformational transitions,^39^ as well as heat-induced aggregation.^40^

Here, building further on that, we demonstrate, by using vT-ESI-MS, its capability to analyze heat induced transitions of covalently and noncovalently linked ADCs. We initially evaluated the method’s ability to recapitulate the stability of a series of well-studied noncovalently linked human IgG4 half molecules^41^ and subsequently applied vT-ESI-MS to characterize a few cysteine- and lysine-linked ADCs. Our findings reveal that different ADCs and their respective DAR-variants behave differently during heat treatment. vT-ESI-MS can effectively capture the stability behavior of different DARs in a single experiment without prior separation.

## Experimental Section

### Sample Preparation

The hinge-deleted human IgG4 anti-epidermal growth factor receptor (EGFR) (IgG4Δhinge) antibodies were recombinantly expressed and purified by Genmab (The Netherlands) and have been described earlier.^41^ 100 μg aliquots of nonmutated “WT”, R409 K, and L368A variants were buffer-exchanged into a 150 mM ammonium acetate solution (pH 7.5) via six iterative cycles of dilution and concentration using 0.5 mL of Amicon Ultra Centrifugal filters (50 kDa MWCO, Merck) at 4 °C. Concentrations were subsequently measured using a NanoDrop Microvolume Spectrophotometer (Thermo Fisher Scientific) at A280, followed by dilution to a final concentration of 2.5 μM, which was subsequently used for vT-ESI native MS.

Three commercially available distinct antibody–drug conjugates (ADCs), namely, trastuzumab emtansine (lysine-linked), enfortumab vedotin, and brentuximab vedotin (both cysteine-linked), were obtained from Evidentic GmbH (Germany). For analysis, 100 μg aliquots of each ADC were diluted in PBS to a concentration of 1 μg/μL. Deglycosylation was performed by adding 10 U and 50 U of *N*-glycosidase F (Roche) to the cysteine- and lysine-linked ADCs, respectively, followed by overnight incubation at 37 °C. Following deglycosylation, the ADCs were buffer-exchanged into a 150 mM ammonium acetate solution, as previously described. The final concentrations were adjusted to the measurable range 1–4 μM to align with subsequent vT-ESI native MS.

### vT-ESI Native Mass Spectrometry

The in-house built vT-ESI source employed in this study was adapted from the design described by McCabe et al.,^36^ incorporating several modifications (Figure S1). In-house fabricated borosilicate nanoemitters (1B120F-4 World Precision Instruments, USA; P-97 Micropipette Puller, Sutter Instruments, USA) were double-coated with a layer of gold using a Scancoat Six Sputter Coater (Edwards Ltd., U.K.). To ensure electrical insulation from the heated brass block, a polyolefin heat-shrink tube (Kai Suh Suh Enterprise, Taiwan) with a 1.5 mm inner diameter was applied over the emitters and subsequently shrunk using a heat gun. The temperature of the brass block was controlled using a TC-720 temperature controller (TE Technology, Inc., USA), which regulated heating and cooling via a four-stage thermoelectric cooler (TEC) model TEC4-97-49-17-7-05 (Conrad Electronic SE, Germany). Temperature readings from a high-accuracy thermistor MP-3189 (TE Technology, Inc., USA) were continuously used as feedback to adjust and stabilize the brass block temperature.

Temperature melting experiments were conducted using an Orbitrap Exactive Plus Extended Mass Range (EMR) mass spectrometer (Thermo Fisher Scientific, Germany). Each emitter was filled with 3 μL of sample, positioned within a brass cone, and inserted into a brass block. The ESI voltage (1.0–1.3 kV) was applied via a conductive sleeve through the gold-coated section of the emitter, followed by a continuous temperature ramp from 30 to 100 °C at a rate of 2 °C/min. MS acquisition parameters were optimized to ensure efficient ion transmission within the 500–10 000 *m*/*z* range (detailed MS parameters are provided in the Table S1).

### Data Analysis

Raw MS data were averaged over a 0.25 min window and deconvoluted using the UniChrom package (part of UniDec, version 7.0.2 ^42^). The processed data were synchronized with the TC-720 temperature readback and visualized by using GraphPad Prism 10.4.1.

## Results

### vT-ESI-MS Recapitulates the Known Stability of Hinge-Deleted and C_H_3-C_H_3-Modified IgG4

To assess whether the in-house built vT-ESI-MS setup would be suitable to study the stability of antibodies and antibody-drug conjugates, we first analyzed a set of hinge-deleted IgG4 antibodies (IgG4Δhinge), as a proof-of-principle system. These IgG4Δhinge antibodies coexist as full noncovalent IgG4 antibodies in equilibrium with their half antibodies,^43^ somewhat reminiscent of fully conjugated cysteine-based ADCs. We selected three IgG4 antibody mutants with distinct and earlier determined *K*_d_ values.^41^ All three antibodies are hinge-modified IgG4s, whereby one has the non-modified, termed “wild-type” (WT), sequence, whereas the two others have a single-point mutation (R409K and L368A, respectively) within the C_H_3–C_H_3 interface. This R409K substitution is a key distinctive difference between natural IgG4 (R) and IgG1–3 sequences (K).^43^ The earlier reported *K*_d_ values for these constructs are 7.55 ± 0.49 μM for L368A (weak binder), 4.97 × 10^–2^ ± 3.1 × 10^–3^ μM for the WT (moderate binder), and 4.03 × 10^–4^ ± 4.4 × 10^–5^ μM for R409K (strong binder). We applied a heat ramp of 2 °C/min and acquired MS1 spectra throughout the heating process. Upon heating of the sample, all three IgG4s dissociate into their respective half-bodies (Figure 1A and Supporting Information Video S1) with different dissociation behaviors that are reflected in their distinct dissociation temperatures (*T*_1/2_) (Figure 1B–D). In accordance with the previously reported *K*_d_ values, the weak binder (L368A) dissociates most easily, with an estimated *T*_1/2_ of 53 °C, followed by the moderate binder (wild type) at 63 °C and last the strong binder (R409K) at 77 °C. While heating the sample, the total signal intensity drops due to aggregation effects (Supporting Information Video S1). As these results agreed with the previously reported data, we determined that the vT-ESI-MS device implemented here could be suitable to assess the heat stability behavior not only of these IgG4Δhinge antibodies but also of other antibodies and ADCs.

**Figure 1:**
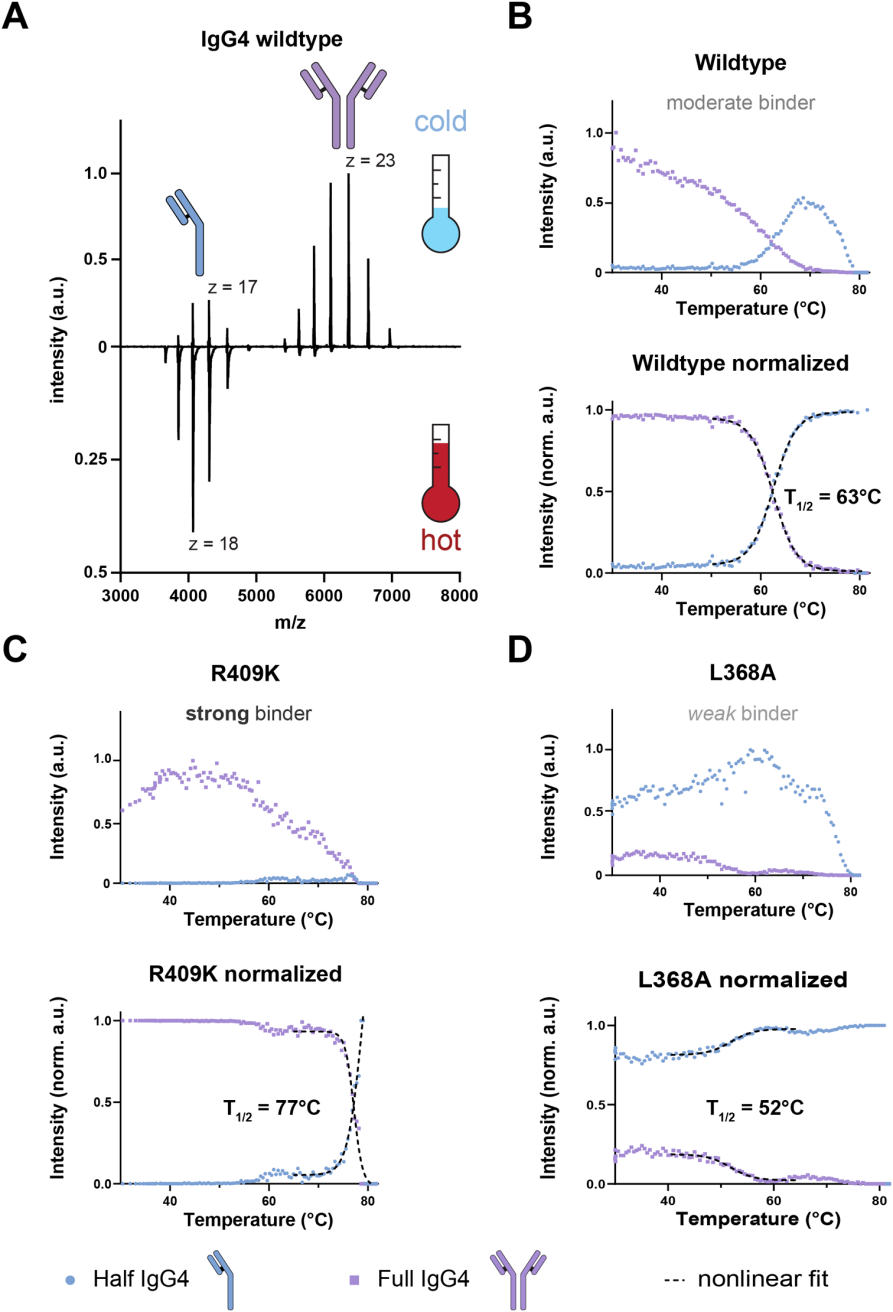
vT-ESI-MS recapitulates the known stability of human hinge-deleted IgG4 antibodies. (A) Comparison of native MS1 spectra of hinge-modified wild-type IgG4 acquired at starting conditions (35 °C, cold) and at ca. 70 °C (hot). The charge envelopes of full and half IgG4 molecules are shown, and the highest intensity charge states (*z*) are indicated. (B) Heat stability profile of hinge-modified wildtype IgG4, depicted as non-normalized (top) and normalized (bottom) data. For normalization, the total sum of the deconvoluted relative intensities of full and half antibodies was set to 1. The full antibody intensity is shown in purple, the half antibody in blue. (C) Heat stability profiles of mutated R409K IgG4, known to be a “strong binder” compared to WT. (D) Heat stability profiles of L368A, known to be a “weak binder” compared to WT. The *T*_1/2_ values determined by vT-ESI-MS are 63, 77, and 52 °C for WT, R409K, and L368A, respectively, in line with their *K*_d_ values 4.97 × 10^–1^, 4.03 × 10^–4^, and 7.55 μM ± SE, respectively.^41^ All curve fit parameters are reported in the Supporting Information (Table S2). Time-resolved native mass spectrometry data are provided in Supporting Information Video S1.

### Cysteine-Linked ADCs Aggregate in a Drug-Load Dependent Manner

As our initial experiments on hinge-deleted IgG4 with different mutations at the C_H_3-C_H_3 interface could faithfully recapitulate the differences in their thermal stabilities, we applied the same workflow to study the behavior of cysteine-linked ADCs brentuximab vedotin and enfortumab vedotin. Both ADCs have been produced with the same linker chemistry, and assuming that the antibody unfolding and aggregation behaviors are similar, we expected to obtain similar results for these two products. Using high-resolution native mass spectrometry,^44^ we were able to mass resolve and detect all 5 different co-occurring DAR species (DARs 0, 2, 4, 6 to DAR 8) of both ADCs (Figure 2B,E) and observed that the drug-load of these two products was alike. Of note, as these are cysteine-linked ADCs, this requires several inter- and intrachain disulfide bridges to be reduced and thus disassembled, as described previously.^30,45^ In the extremes, this renders the DAR 0 product potentially fully covalently assembled by intact disulfide bridges, whereas in DAR 8 the heavy and light chain are only held together by noncovalent interactions. Next, we applied a heat ramp of 2 °C/min and acquired MS1 spectra throughout the heating process. Both ADCs exhibited similar heat stability behavior; namely, they aggregated, albeit in a DAR-dependent manner (Figure 2A,C,D,F). While all DAR species (DARs 0–8) decreased in intensity when heat was applied, a higher drug load (e.g., DAR 6 and DAR 8) leads to a higher tendency to aggregate already at lower temperatures. To illustrate, the fully occupied DAR 8 ADC is not measurable anymore at temperatures above 60 °C; DAR 6 is not measurable at temperatures above 70 °C. DARs 0, 2, and 4 are still detectable up to nearly 80 °C until they also disappear and aggregate completely. While heating the ADCs, we notice a slight shift in charge states, which can be explained by increased flexibility and structural unfolding at higher temperatures (Figure 2A,D). Partial refolding during the ionization process might contribute to the measurable degree of unfolding. As noted previously,^38^ the analyte can cool down and refold as it travels between the heated emitter and the mass spectrometer inlet. At temperatures above 80 °C, we could also detect the ADC light chains with conjugated drug molecules, which only can originate from the higher DAR antibodies, that harbor noncovalent heavy- and light-chain interactions, as the disulfide bonds have been disassembled. To calculate the *T*_1/2_ values of the cysteine-linked ADCs, we normalized the intensities (intensity at 40 °C is 1) and fit a four-parameter nonlinear regression model. The calculated *T*_1/2_ values for the best fit of DARs 8, 6, 4 of brentuximab vedotin were 47, 51, and 56 °C, respectively. For enfortumab vedotin, they were 47, 50, and 52 °C, respectively. The same model did not provide sufficient accuracy for the lower DAR species; hence, we omitted them. However, it was clear from the data that they have higher *T*_1/2_ values when compared to DARs 8, 6, 4 (Figure 2C,G,H). Thus, there is a clear trend observed in the thermal stability of the cysteine-coupled ADCs; the higher is the drug load, the lower are the observed *T*_1/2_ values, for both brentuximab vedotin and enfortumab vedotin.

**Figure 2:**
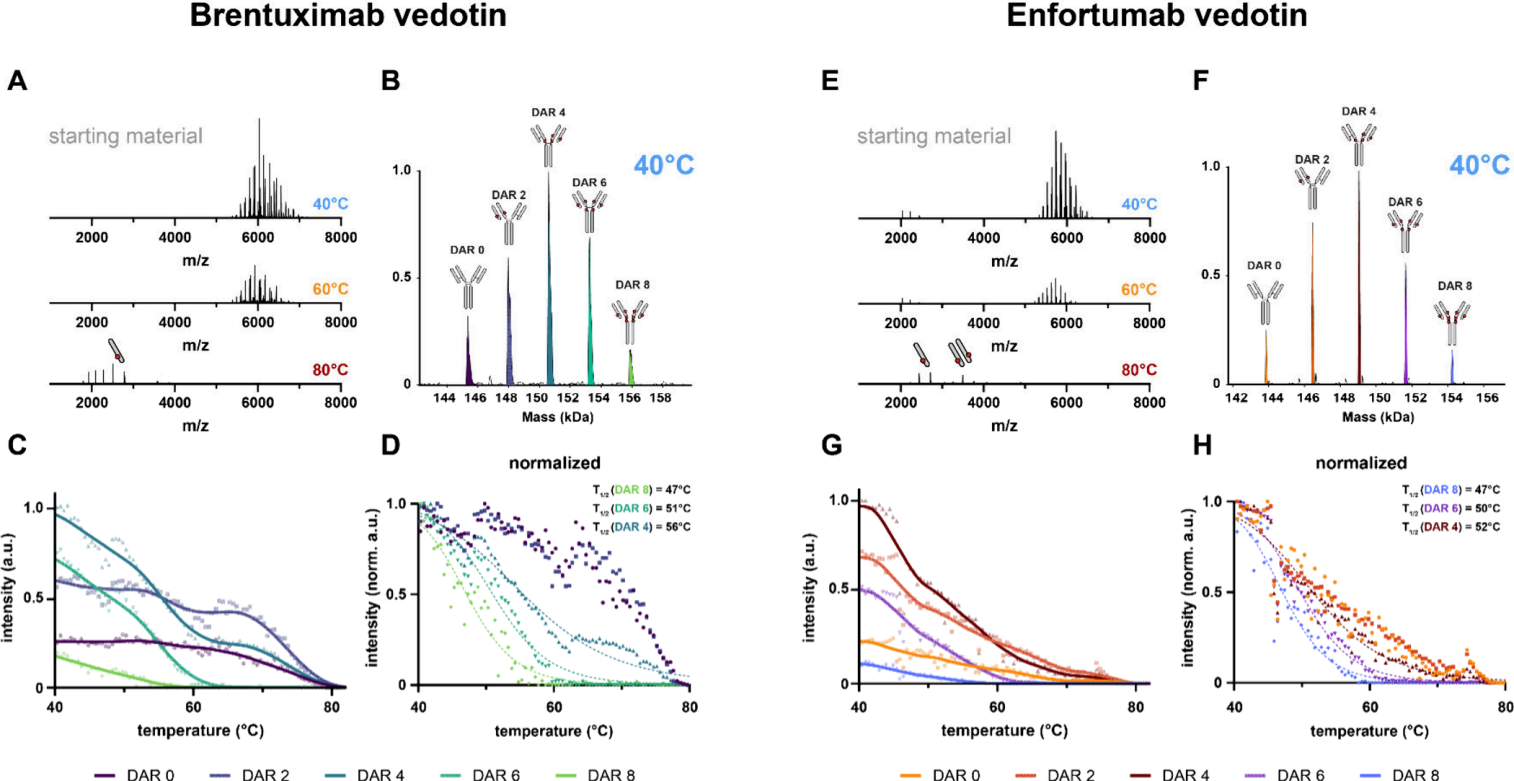
Cysteine-linked ADCs show DAR-dependent heat stability profiles. (A) Raw MS1 spectra of brentuximab vedotin at different temperatures. (B) Deconvoluted mass spectrum of brentuximab vedotin, at 40 °C, with the drug loads indicated. (C) Heat stability profiles of the distinct brentuximab vedotin DAR species. (D) Normalized heat stability profiles of the distinct brentuximab vedotin DAR species, including nonlinear regression for DARs 4, 6, and 8 and calculated *T*_1/2_ values (47 °C for DAR 8, 51 °C for DAR 6, and 56 °C for DAR 4). The intensities were scaled between 1 and 0, with 1 being the highest intensity and 0 being the lowest. (E) Raw MS1 spectra of enfortumab vedotin at different temperatures. (F) Deconvoluted mass spectrum of enfortumab vedotin, at 40 °C, with the drug loads indicated. (G) Heat stability profiles of the distinct brentuximab vedotin DAR species. (H) Normalized heat stability profiles of the distinct enfortumab vedotin DAR species, including nonlinear regression for DARs 4, 6, and 8 and calculated *T*_1/2_ values (47 °C for DAR 8, 50 °C for DAR 6, and 52 °C for DAR 4). All curve fit parameters are reported in the Supporting Information (Table S3).

### Lysine-Linked ADCs Aggregate Independently of Their Drug-Load

To test whether the DAR-dependent heat stability behavior of cysteine-linked ADCs is solely a consequence of the disruption of disulfide bridges or also related to the drug-load itself, we next evaluated the heat stability behavior of trastuzumab emtansine, a lysine-linked ADC. We were able to mass resolve, detect, and quantitatively monitor DARs 0–6 (Figure 3A,B), and even some small amounts of DAR 7. The native mass spectra of trastuzumab emtansine were notably more complex than the spectra of the cysteine-linked ADCs, mainly due to the presence of the unoccupied linker MCC (Figure 3B). This resulted in additional observed peaks with a mass difference of 220–230 Da (MCC = 220 Da). When applying heat to trastuzumab emtansine, all DAR species decreased in intensity and aggregated uniformly and were not detectable anymore at around 85 °C (Figure 3C). This behavior is quite distinct from the cysteine-linked ADCs, and we hypothesize that this is due to the fact that the disulfide bonds in this lysine-linked ADC are still intact. To assess *T*_1/2_ for this ADC, we normalized the measured intensities (intensity at 40 °C is 1) and fit a linear regression model onto the measured intensities (Figure 3D). *T*_1/2_ was calculated at *y* = 0.5. The calculated *T*_1/2_ values for the best linear fit for DAR 0, DAR 1, DAR 2, DAR 3, DAR 4, DAR 5, and DAR 6 were 68, 66, 66, 65, 64, 63, and 61 °C, respectively (Table S4). These values are significantly higher compared to the *T*_1/2_ values of the measured cysteine-linked ADCs. However, they also show DAR-dependent differences, albeit of lower absolute difference. Interestingly, the heat stability behavior and the decline in intensity could not be described with a four-parameter sigmoidal model for the higher DAR species of cysteine-linked ADCs and for the hinge-deleted IgG4. Instead, the behavior more closely resembled that of the lower DAR species of cysteine-linked ADCs. This leads us to hypothesize that the disulfide linkage is the main contributor to the heat stability differences that we can measure between cysteine- and lysine-linked ADC products.

**Figure 3:**
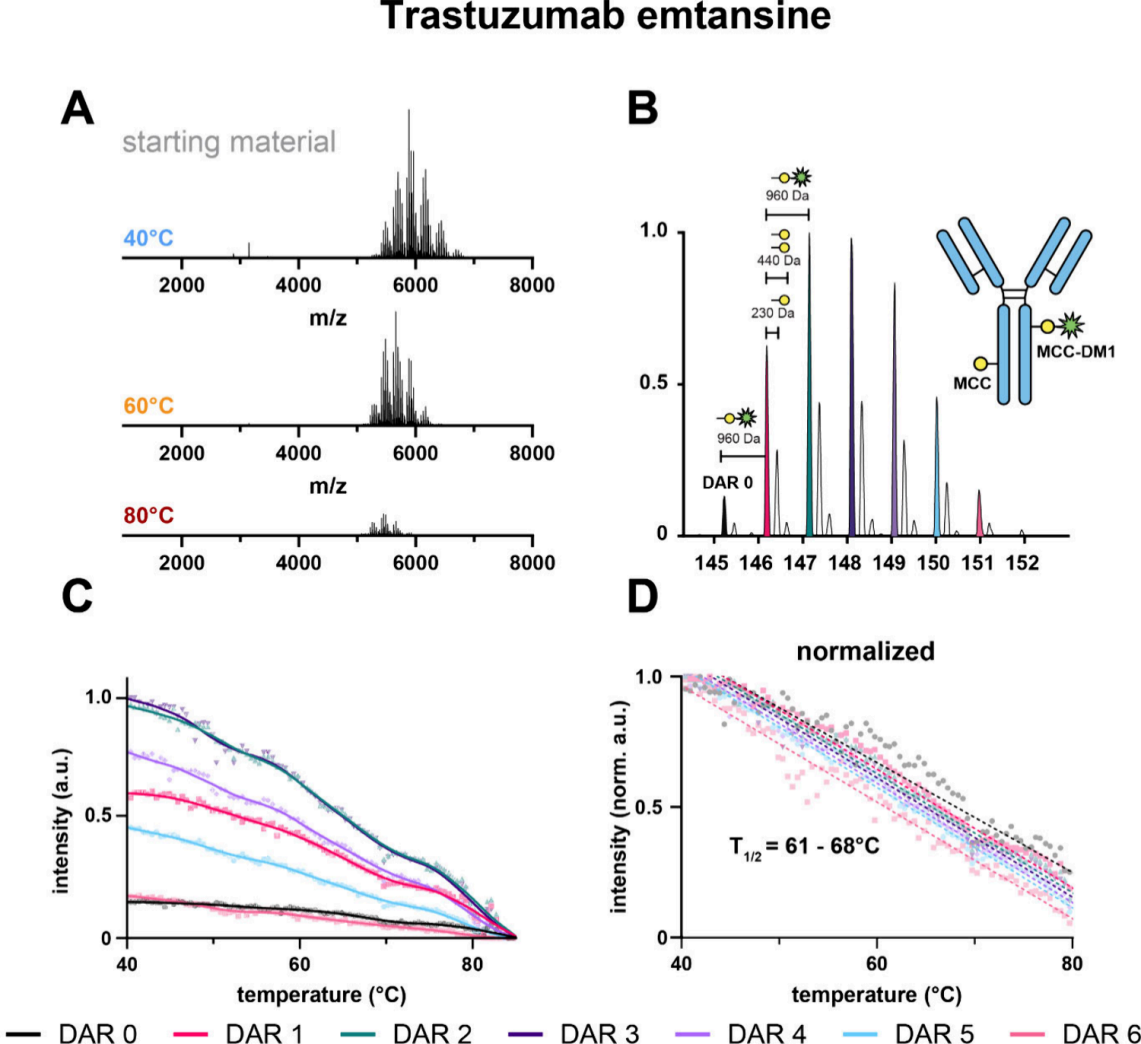
Lysine-linked ADC trastuzumab emtansine shows a drug load independent heat stability behavior. (A) Raw MS1 spectra of trastuzumab emtansine at different temperatures. (B) Deconvoluted native mass spectrum of trastuzumab emtansine. (C) Heat stability profiles of the detected trastuzumab emtansine DAR species. (D) Normalized heat stability profiles (maximum intensity of each species = 1), including linear regression of all profiles. *T*_1/2_ was calculated at *y* = 0.5. The calculated *T*_1/2_ values for the best linear fit for DAR 0, DAR 1, DAR 2, DAR 3, DAR 4, DAR 5, and DAR 6 were 68, 66, 66, 65, 64, 63, and 61 °C, respectively. All curve fit parameters are reported in the Supporting Information (Table S4). The intensities were scaled between 1 and 0, with 1 being the highest intensity for each species and with 0 being the lowest.

## Discussion

It has been stated that antibody–drug conjugates (ADCs) represent a transformative advancement in cancer therapy, combining the precision of monoclonal antibodies with the potency of cytotoxic drugs to target cancer cells while sparing healthy tissue.^46^ Notwithstanding this tribute, it should not be overlooked that antibody–drug conjugates are mostly heterogeneous biotherapeutic mixtures of multiple positional isomers (see Figure 4). As comprehensively reviewed by Walsh et al.,^45^ several key factors have to be considered for the successful development of ADCs: (1) the conjugation has to be stable in circulation, (2) the drug-load has to be optimized to ensure optimal efficacy with optimal safety, (3) the drug attachment should not affect target recognition and binding, and (4) the conjugation reaction should allow for a controlled and consistent modification of antibodies to generate ADCs. The attached cytotoxic drugs are typically hydrophobic, leading to an increased aggregation propensity with increased site occupancy.

**Figure 4:**
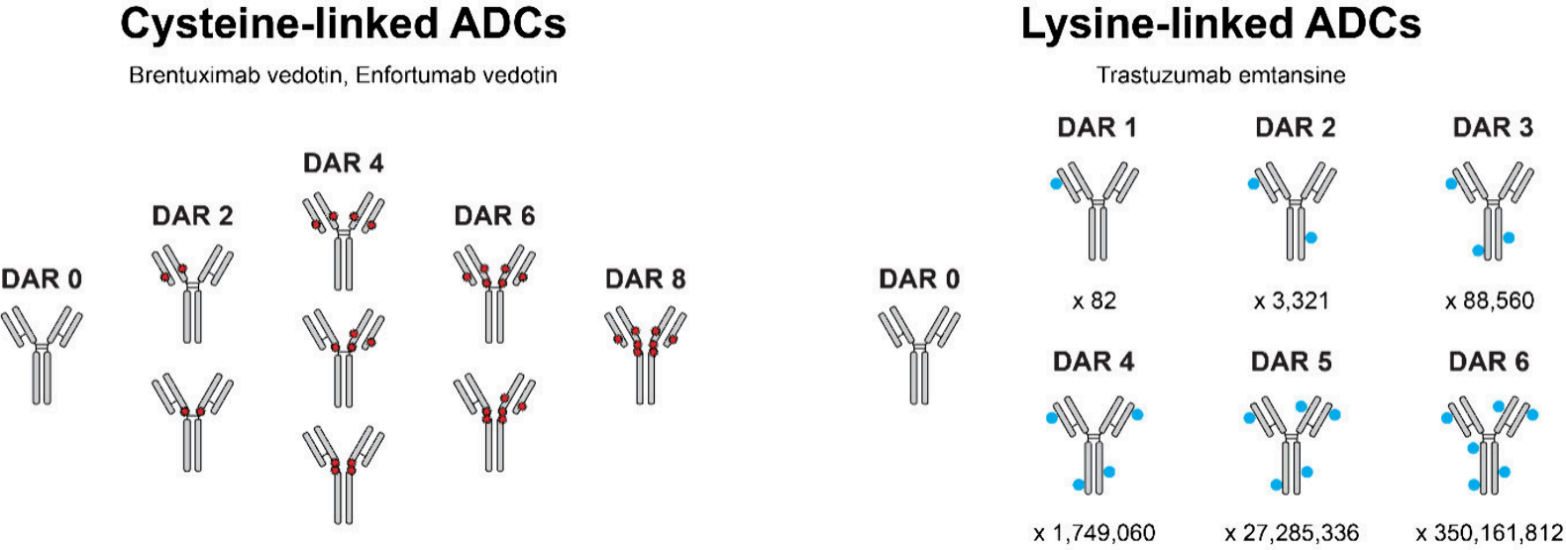
ADCs represent often heterogeneous biotherapeutics of multiple positional isomers. (Left) Positional isomers of the cysteine-linked ADCs (note that either the cysteines at the top or bottom hinge position can be modified, resulting in 3 additional positional isomers). (Right) positional isomers of the lysine-linked ADC up to DAR 6 with the theoretical number of positional isomers that could originate from the previously reported accessible lysine sites (82 ^47^). Due to steric hindrance, reactivity differences, and preferential modification, which heavily influence the site occupancy, the experimental number will likely be significantly lower. Theoretical positional isomers were calculated as $C_{k}\left(n\right)=\left(\begin{array}{c} n\\ k \end{array}\right)=\frac{n!}{k!\left(n-k\right)!}$ with *n* being the number of possible modification sites and *k* the number of occupied sites.

Of particular interest, cysteine-linked ADCs are modified via mild reduction of their 4 interchain disulfides, resulting in 9 positional isomers (12 isomers if both hinge-region disulfides are considered). Lysine-linked ADCs can even be more heterogeneous as they can theoretically be modified at every free amine group (N-terminus or lysine) in the ADC. Previous studies have shown that the lysine-linked ADC trastuzumab emtansine contains 92 possible linkage sites (4 N-terminal amines, 88 lysines), of which up to 82 are partially occupied, with some “hot spots”^48^ and 3 sites that could be occupied with just the non-drug-conjugated MCC linker.^47,49^ Since higher DARs lead to compromised structural integrity, the payloads, as well as their stabilities, need to be carefully controlled and should typically remain below ∼8. Although efforts have been made to design ADCs with well-defined payloads at specific sites in the mAb, the clinically approved and used ADCs are still all mixtures of positional isomers, whose stability and reactivity may not be identical.

Here, we explore variable temperature electrospray ionization mass spectrometry to monitor the thermal stability of structural isomers of ADCs with specific drug-loads. First, we show that the in-house constructed vT-ESI-MS setup works well and can be used to study the heat stability of various modified monoclonal IgG4 antibodies. In a proof-of-principle experiment, we demonstrated that vT-ESI-MS faithfully recapitulates the stability of IgG4 antibodies with mutations in the C_H_3–C_H_3 region. Based on this proof of concept, we next applied vT-ESI-MS to characterize the heat stability of antibody–drug conjugates.

We investigated the thermal stability of a few clinically approved cysteine- and lysine-linked ADCs and showed that the linker chemistry and occupancy profoundly influence the stability of the final ADC drug product. In particular, we studied the two cysteine-linked ADCs brentuximab vedotin (Adcetris) and enforumab vedotin (Padcev), as well as the lysine-linked ADC trastuzumab emtansine (Kadcyla). The cysteine-linked ADCs are coupled to the cytotoxic drug monomethyl auristatin E (MMAE) via a maleimide attachment group and a cathepsin cleavable linker. Trastuzumab emtansine, on the other hand, is coupled to the cytotoxic drug DM1 linked through the heterobifunctional linker succinimidyl 4-(*N*-maleimidomethyl)­cyclohexane-1-carboxylate (SMCC). The succinimidyl group of SMCC is used for the coupling to lysine residues, and the maleimide group is used for the coupling of DM1. This notable difference in linker chemistries gives rise to the varying drug-loads that are observed in cysteine- and lysine-linked ADCs. We revealed that the studied cysteine-linked ADCs exhibited drug-load-dependent melting behavior, with higher DAR species aggregating first, followed by species containing a lower drug load. This behavior, we hypothesize, can be best explained by the partial lack of interchain disulfide bridges that normally connect the two half an tibodies. We observed similar results with hinge-deleted IgG4 antibodies. These IgG4 antibodies are somewhat similar to highly occupied cysteine-linked ADCs, as they are also solely held together by noncovalent interactions between the half antibodies. Upon heating, they disassemble and aggregate, dependent on the amino acids at the C_H_3–C_H_3 interface.

Remarkably, the heat stability behavior of DAR 8 and DAR 6 of the cysteine-linked ADCs can be described with sigmoidal models (Figure 2D,H), similar to the behavior of the analyzed C_H_3–C_H_3-interface-modified IgG4s (Figure 1B–D). DAR 4 can still be described with a sigmoidal model but less accurately, possibly related to the increased number of positional isomers (Figure 4). DAR 2 and DAR 0 cannot be described with this model. We hypothesize that this behavior has to do with (1) the noncovalent interactions that hold together the higher DAR species and (2) the number of positional isomers. While we could follow the disassembly of IgG4 due to the detectability of half-bodies, we do not observe the presence of ADC half-bodies in our experiments, likely due to rapid aggregation after disassembly. We were able to detect a shift in charge states, indicative of protein unfolding. However, the cooling of the analyte as it travels from the heated emitter to the mass spectrometer during the process of ionization can lead to refolding and might limit the degree of measurable unfolding.^38^ This cooling effect could be minimized by using either a heat shield as demonstrated by Wang et al.^38^ or a setup that heats the entire source region between the emitter and the mass spectrometer inlet as described by Daneshfar et al.^50^

The heat stability behavior of the lysine-linked ADC trastuzumab emtansine differed greatly from that of the cysteine-linked ADCs, as its melting behavior could be described with a linear regression model. While the differences in *T*_1/2_ were notably smaller between the different DAR species, they were still somewhat DAR-dependent (Figure 3D). The normalized data showed that the highly occupied species have lower *T*_1/2_ values the less occupied species. Previous studies have shown that lysine linkage can destabilize the C_H_2 domain and leads to higher propensity for aggregation,^51^ which is in agreement with the current observations.

## Conclusion

The design of optimal ADCs with high potency and stability requires careful optimization of the specific drug-loads. Like previous studies, our data show that increased DARs lead to higher aggregation propensities. Therefore, we suggest that the heat stability behavior of individual DAR species should be considered and closely monitored during drug development as this can influence the effectively delivered drug dose. In such analyses, vT-ESI-MS could be applied as a method to study not only the influence of the linker chemistry on the overall heat stability but also how the drug-load affects the stability, a feature that is often missed in bulk stability measurements.

## Supplementary Material





## Data Availability

The mass spectrometry data underlying this study are openly available and have been deposited to the ProteomeXchange Consortium via the PRIDE partner^52^ repository with the data set identifier PXD063887.

## Notes

Video S1 showing the thermal unfolding and native mass spectrometry analysis of wild-type IgG4 and mutants (MP4)
Details on the design of the variable temperature source; mass spectrometry measurement parameters; curve and linear fit parameters for the different ADCs (PDF)
