# Supplementary material for: Antibody–Drug Conjugate Stability Probed by Variable-Temperature Electrospray Ionization Mass Spectrometry
Source: J Am Soc Mass Spectrom. 2025 May 23;36(6):1395–403. doi: 10.1021/jasms.5c00109 (PMC12142670; doi:10.1021/jasms.5c00109)
Supplement: Supplementary file 2 [file js5c00109_si_002.pdf]

Supplementary information for:

Antibody-drug conjugate stability probed by variable-temperature electrospray ionization mass spectrometry

Jan Fiala[1,2,#], Dina Schuster[1,2,#], Albert J. R. Heck[1,2,*]

[1]Biomolecular Mass Spectrometry & Proteomics, Bijvoet Center for Biomolecular Research & Utrecht Institute for Pharmaceutical Sciences, Utrecht University, Padualaan 8, 3584 CH Utrecht, The Netherlands.

[2]Netherlands Proteomics Center, Padualaan 8, 3584 CH Utrecht, The Netherlands

[#]contributed equally *corresponding author: a.j.r.heck@uu.nl

Supplementary Figure S1. Variable temperature source

Supplementary Table S1. Mass spectrometer parameters for vT-ESI experiments

Supporting Movie S1: Thermal unfolding and native mass spectrometry analysis of wild-type IgG4 and mutants

Supplementary Table S2. Curve fit parameters for hinge-deleted IgG4

Supplementary Table S3. Curve fit parameters for cysteine ADCs (DAR 4 - DAR 8)

Supplementary Table S4. Linear fit parameters for lysine ADC (DAR 0 – DAR 6)

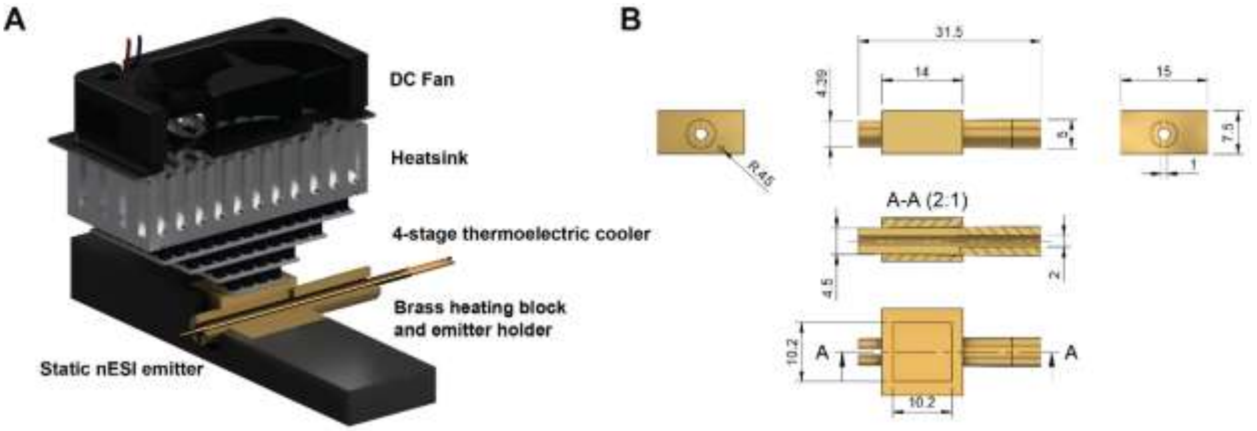

Supplementary Figure S1. Variable temperature source A) 3D design of the variable-temperature-controlled nanoESI source used in this work, inspired by the design of McCabe *et al.* [1]. The current design incorporates components with a reduced footprint compared to the referenced work, enabling direct compatibility with the Nanospray Flex Ion Source (Thermo Scientific) without requiring hardware modifications. B) Technical drawing of the brass block used for heating the sample in the nanoESI emitter via a four-stage thermoelectric cooler (TEC).

Supplementary Table S1. Mass spectrometer parameters for vT-ESI experiments

Mass spectrometer parameter	IgG4	ADCs
Scan range (m/z)	500-15 000	500-15 000
Resolution (@m/z 400)	35 000	17 500
Microscans	10	10
Injection time (ms)	10	10
Capillary temperature (°C)	200	170
S-Lens RF level	120	200
S-Lens voltage (V)	50	25
Injection flatapole DC (V)	10	10
Inter-flatapole DC (V)	7	7
Bent flatapole DC (V)	2	5
Transfer multipole DC (V)	2	4
HCD direct eVenergy (eV)	90	50-120
Trapping gas settings	3	3

Supporting Movie S1: Thermal unfolding and native mass spectrometry analysis of wild-type IgG4 and mutants. Time-resolved native mass spectrometry coupled to a thermal ramp was used to assess the thermal stability of wild-type IgG4 (A), L368A (weak binder, B), and R409K (strong binder, C). Top panels show total ion intensity (blue trace) overlaid with the set and measured temperature gradient (red and blue line). The $T_{1/2}$ values, indicating the midpoint of thermal unfolding, are marked for each variant. Wild-type IgG4 shows a $T_{1/2}$ of 63 °C, L368A unfolds earlier at 53 °C, and R409K demonstrates increased thermal stability with a $T_{1/2}$ of 77 °C. The corresponding mass spectra (bottom panels) show the detected species in the 2 min averaged windows, with highlighted peaks corresponding to half (blue) and full IgG4s (purple).

Supplementary Table S2. Curve fit parameters for hinge-deleted IgG4

		**Best fit**		**95% confidence interval**	
Wildtype		**Halfbody**	**Full IgG4**	**Halfbody**	**Full IgG4**
	Bottom	0.05068	0.01154	0.03992 to 0.06113	0.004410 to 0.01859
	Top	0.9865	0.9498	0.9785 to 0.9946	0.9395 to 0.9604
	$T_{1/2}$	62.53	62.55	62.41 to 62.66	62.43 to 62.66
	Slope	2.285	-2.299	2.177 to 2.399	-2.409 to -2.195
	R^2	0.9984	0.9985		
R409K		**Best fit**		**95% confidence interval**	
		**Halfbody**	**Full IgG4**	**Halfbody**	**Full IgG4**
	Bottom	0.05371	-0.01648	0.03196 to 0.07358	-0.04217 to 0.008565
	Top	1.899	0.933	1.272 to 9.320	0.9143 to 0.9519
	$T_{1/2}$	78.86	77.16	77.72 to 83.26	77.00 to 77.32
	Slope	1.477	-0.8741	1.105 to 2.057	-1.020 to -0.7442
	R^2	0.9819	0.991		
L368A		**Best fit**		**95% confidence interval**	
		**Halfbody**	**Full IgG4**	**Halfbody**	**Full IgG4**
	Bottom	0.813	0.02259	0.8013 to 0.8227	0.01357 to 0.03067
	Top	0.9774	0.187	0.9693 to 0.9864	0.1773 to 0.1987
	$T_{1/2}$	51.81	51.81	51.14 to 52.45	51.14 to 52.45
	Slope	2.185	-2.185	1.691 to 2.823	-2.823 to -1.691
	R^2	0.9618	0.9618		

Supplementary table S2. Curves were fit in GraphPad 10.4.1, using a non-linear, sigmoidal regression model according to $Y = Bottom + \frac{(Top-Bottom)}{1+e^{\frac{T_{1/2}-X}{Slope}}}$. Best fits and 95% confidence intervals are reported accordingly.

Supplementary Table S3. Curve fit parameters for cysteine ADCs (DAR 4 - DAR 8)

Brentuximab vedotin	**Best fit**			
		**DAR 4**	**DAR 6**	**DAR 8**
	$T_{1/2}$	55.51	50.99	47.12
	HillSlope	-8.175	-12.41	-13.17
	R^2	0.9782	0.9758	0.9565
	**95% Confidence Interval**			
	$T_{1/2}$	55.09 to 55.92	50.66 to 51.32	46.78 to 47.46
	HillSlope	-8.632 to -7.751	-13.26 to -11.62	-14.27 to -12.17
Enfortumab vedotin	**Best fit**			
		**DAR 4**	**DAR 6**	**DAR 8**
	$T_{1/2}$	51.61	49.76	47.17
	HillSlope	-9.562	-13.05	-14.04
	R^2	0.9521	0.9714	0.9476
	**95% Confidence Interval**			
	$T_{1/2}$	51.05 to 52.18	49.39 to 50.14	46.74 to 47.59
	HillSlope	-10.48 to -8.738	-14.18 to -12.04	-15.70 to -12.59

Supplementary table S3. Curves were fit in GraphPad 10.4.1, using a non-linear, sigmoidal regression model according to $Y = Bottom + \frac{Top-Bottom}{1+\frac{X}{T_{1/2}}}$, where Top = 1 and Bottom = 0. Best fits and 95% confidence intervals are reported accordingly.

Table S4. Linear fit parameters for lysine ADC (DAR 0 – DAR 6)

	Best fit							
		DAR 0	DAR 1	DAR 2	DAR 3	DAR 4	DAR 5	DAR 6
Trastuzumab emtansine	Slope	-2.102	-2.268	-2.279	-2.285	-2.288	-2.303	-2.231
	$Y_{intercept}$	193.2	200.6	199.6	198.4	196.5	195.8	185.8
	R^2	0.9329	0.9781	0.9842	0.9847	0.9904	0.9935	0.9808
	$T_{1/2}$	68.13	66.40	65.64	64.95	64.03	63.31	60.87
	95% Confidence Interval							
	Slope	-2.219 to -1.984	-2.338 to -2.197	-2.339 to -2.219	-2.344 to -2.226	-2.335 to -2.241	-2.342 to -2.265	-2.296 to -2.166
	$Y_{intercept}$	185.6 to 200.8	196.0 to 205.2	195.7 to 203.5	194.5 to 202.2	193.5 to 199.5	193.3 to 198.3	181.6 to 190.0

Supplementary table S4. Curves were fit in GraphPad 10.4.1, using a basic linear regression model according to $Y = Slope * X + Y_{intercept}$. $T_{1/2}$ was calculated as $T_{1/2} = \frac{50 - Y_{intercept}}{Slope}$. Best fits and 95% confidence intervals are reported accordingly.

References

1. McCabe, J. W.; Shirzadeh, M.; Walker, T. E.; Lin, C. W.; Jones, B. J.; Wysocki, V. H.; Barondeau, D. P.; Clemmer, D. E.; Laganowsky, A.; Russell, D. H., Variable-Temperature Electrospray Ionization for Temperature-Dependent Folding/Refolding Reactions of Proteins and Ligand Binding. *Anal Chem* **2021,** *93* (18), 6924-6931.
